# Supplementary material for: The genome sequence of an ichneumonid wasp, Ephialtes manifestator (Linnaeus, 1758)
Source: Wellcome Open Res. 2024 Aug 12;9:451. [Version 1] doi: 10.12688/wellcomeopenres.22775.1 (PMC12475899; doi:10.12688/wellcomeopenres.22775.1)
Supplement: Peer review history

# Reviewer report: Reviewer response for version 1

**DOI:** 10.21956/wellcomeopenres.25079.r133481

**Published:** 2025-09-26

**Authors:** Mostafa Ghafouri Moghaddam

**Affiliations:** 1 Chulalongkorn University Press, Bangkok, Bangkok, Thailand; 2 Department of Biology, Chulalongkorn University (Ringgold ID: 176057), Bangkok, Bangkok, Thailand

The manuscript presents a chromosomally complete genome assembly of *Ephialtes manifestator* (Ichneumonidae: Pimplinae). Using PacBio HiFi long-read sequencing and Hi-C scaffolding, the authors assembled a 577.70 Mb genome, most of which (95.31%) was scaffolded into 15 chromosomal pseudomolecules. The mitochondrial genome (30.82 kb) was also assembled. The work provides a valuable genomic resource for future studies on ichneumonid systematics, phylogeny, and parasitoid-host interactions.

 This is a clear, well-written, and scientifically sound contribution. The work is of high technical quality, consistent with the Darwin Tree of Life standards, and provides an important new genomic resource for Hymenoptera research. The data will be of interest not only to genome biologists but also to systematists and ecologists working on parasitoid–host interactions.

1. The abstract is concise and accurate but could briefly emphasize the biological importance of sequencing *E. manifestator* (e.g., its relevance to ichneumonid systematics, parasitoid biology, or underrepresentation in genomic studies). This would help contextualize the technical achievements.
2. The background section is clear, but a short expansion would help non-specialists. For instance, outlining why ichneumonid genomes are scarce, or how this assembly could contribute to resolving phylogenetic relationships within Ichneumonidae, would better highlight the manuscript’s significance.
3. The figures are appropriate and informative. To make them more accessible, the legends could briefly explain terms such as “snail plot” or “scaffold N50,” which may not be familiar to all readers.

While not essential, a short comparison with existing ichneumonid genomes (if available) would help place this assembly in context, underscoring its novelty.

 Accept with minor suggestions!

Are sufficient details of methods and materials provided to allow replication by others?

Yes

Is the rationale for creating the dataset(s) clearly described?

Yes

Are the datasets clearly presented in a useable and accessible format?

Yes

Are the protocols appropriate and is the work technically sound?

Yes

Reviewer Expertise:

Evolutionary biology (phylogenetics and phylogenomics of parasitoid wasps)

I confirm that I have read this submission and believe that I have an appropriate level of expertise to confirm that it is of an acceptable scientific standard.

# Reviewer report: Reviewer response for version 1

**DOI:** 10.21956/wellcomeopenres.25079.r100526

**Published:** 2024-09-24

**Authors:** Christopher Wyatt

**Affiliations:** 1 University College London, London, England, UK

*Ephialtes manifestator* is from the Pimplinae, which is an understudied family of parasitoid wasps. This chromosome level genome provides the first published genome of this species to my knowledge. The length of the genome is consistent with previously published parasitoids, with comparable contiguity to other wasps (N50) and similar number of chromosomes. This provides a great resource for the community for study of this species.

Are sufficient details of methods and materials provided to allow replication by others?

Yes

Is the rationale for creating the dataset(s) clearly described?

Yes

Are the datasets clearly presented in a useable and accessible format?

Yes

Are the protocols appropriate and is the work technically sound?

Yes

Reviewer Expertise:

genomics, transcriptomics, evolutionary biology

I confirm that I have read this submission and believe that I have an appropriate level of expertise to confirm that it is of an acceptable scientific standard.
